# Proteasome inhibition boosts autophagic degradation of ubiquitinated-AGR2 and enhances the antitumor efficiency of bevacizumab

**DOI:** 10.1038/s41388-019-0675-z

**Published:** 2019-01-15

**Authors:** Dawei Wang, Qingqing Xu, Quan Yuan, Mengqi Jia, Huanmin Niu, Xiaofei Liu, Jinsan Zhang, Charles Yf Young, Huiqing Yuan

**Affiliations:** 1 Key Laboratory of Experimental Teratology of the Ministry of Education, Institute of Medical Sciences, The Second Hospital of Shandong University, Jinan, China; 2 Department of Urology, Mayo Clinic College of Medicine, Mayo Clinic, Rochester, MN USA

**Keywords:** Cancer therapeutic resistance, Ubiquitylation, Cancer therapeutic resistance, Ubiquitylation

## Abstract

Anterior gradient 2 (AGR2), a protein belonging to the protein disulfide isomerase (PDI) family, is overexpressed in multiple cancers and promotes angiogenesis to drive cancer progression. The mechanisms controlling AGR2 abundance in cancer remain largely unknown. Here, we observed that AGR2 expression is significantly suppressed by proteasome inhibitor MG132/bortezomib at mRNA and protein levels in lung cancer cells. MG132-mediated repression of AGR2 transcription was independent of ROS generation and ER stress induction, but partially resulted from the downregulated E2F1. Further investigation revealed that MG132 facilitated polyubiquitinated AGR2 degradation through activation of autophagy, as evidenced by predominant restoration of AGR2 level in cells genetic depletion of Atg5 and Atg7, or by autophagy inhibitors. Activation of autophagy by rapamycin noticeably reduced the AGR2 protein in cells and in the mouse tissue samples administrated with bortezomib. We also provided evidence identifying the K48-linked polyubiquitin chains conjugating onto K89 of AGR2 by an E3 ligase UBR5. In addition, an autophagy receptor NBR1 was demonstrated to be important in polyubiquitinated AGR2 clearance in response to MG132 or bortezomib. Importantly, downregulation of AGR2 by proteasome inhibition significantly enhanced antitumor activity of bevacizumab, highlighting the importance of AGR2 as a predictive marker for selection of subgroup patients in chemotherapy.

## Introduction

Human anterior gradient 2 (AGR2), a member of the protein disulfide isomerase (PDI) family, is firstly identified as differentially expressed in estrogen receptor-positive breast cancer cells [1]. Growing evidence has been demonstrated that AGR2 is overexpressed in prostate cancer [2], lung cancer [3], breast cancer [4], and pancreatic cancer [5], and promotes cell proliferation, invasion, and metastasis via multiple mechanisms [4–7]. In addition, changes in the expression level of AGR2 have been observed in response to chemotherapeutic agents including docetaxel and tamoxifen. These findings suggest that AGR2 might be a potential tumor biomarker that predicts the response to therapy [8, 9]. Recently, an interaction of AGR2/VEGF was demonstrated to contribute to the pro-metastatic activity of AGR2 on angiogenesis in ours and Guo’s study [10, 11]. We provided evidence that extracellular AGR2 impairs anti-angiogenesis and antitumor efficiency of bevacizumab. Given the important roles of AGR2 in conferring a metastatic phenotype and increasing chemoresistance when overexpressed in cancers, efforts have been made to investigate the regulatory mechanisms controlling expression of the AGR2, and develop strategies to block AGR2 activity. For example, AGR2 transcription is positively regulated by estrogen and androgen [12]. AGR2 is also up-regulated upon ER stress to maintain ER homeostasis, mainly resulting from activation of the ATF6 and IRE1 signaling pathways of the UPR [13]. However, anti-estrogen tamoxifen, a drug attenuates estrogen receptor function, induces AGR2 expression, which in turn conferring drug resistance [14]. Recent published work shows that AGR2 blocking antibody can suppress its pro-metastatic binding partner VEGF, displaying the potential as a therapeutic agent [11].

In the present study, we aimed to delineate the mechanisms that facilitate AGR2 degradation, leading to the enhanced effectiveness of cancer therapy. After screening agents that are clinically used, proteasome inhibitor MG132/bortezomib is identified to have ability to decreases AGR2 expression at both mRNA and protein levels in an ER stress-independent manner. Activation of autophagy by proteasome inhibition facilitates the degradation of polyubiquitinated AGR2 protein. Moreover, suppression of AGR2 by bortezomib significantly enhances antitumor efficiency of bevacizumab, an angiogenesis-targeting therapeutic drug.

## Results

### Inhibition of proteasome decreases the AGR2 both at mRNA and protein levels

**Fig. 1:**
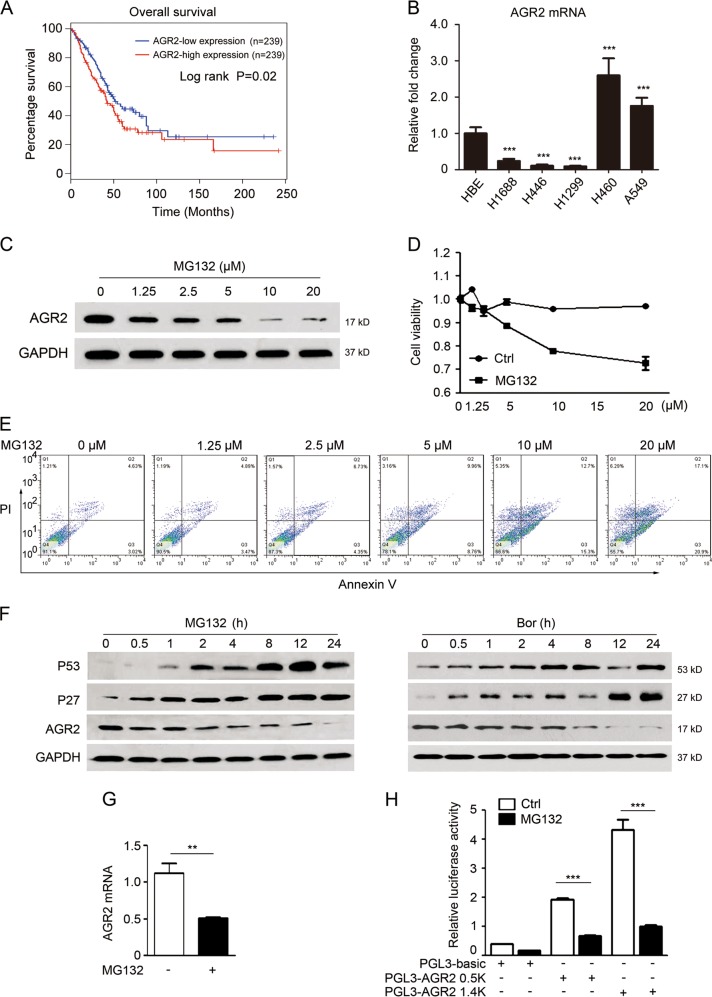
Proteasome inhibition decreases the AGR2 expression. **a** Kaplan–Meier survival curves of lung adenocarcinoma patients are plotted for AGR2. The survival rate of the patients in the AGR2-lower group was significantly higher than that of patients in the AGR2-higher group (log-rank test, *p*= 0.02). **b** The baseline expression of AGR2 in several lung cancer cell lines. GAPDH served as a loading control. **p < 0.01 and ***p < 0.001 compared with the HBE cells. **c** The expression change of AGR2 in MG132-treated A549 cells was estimated by western blotting analysis. **d** MTT assay and **e** annexin staining was used to evaluate the effect of MG132 on A549 cells at 24 h. All values are expressed as the means ± SD of three independent experiments. **f** The levels of p53, p27, and AGR2 were measured by western blotting with 5 μM MG132 or 10 μM bortezomib for indicated time. **g** QRT-PCR analysis of AGR2 mRNA levels with 5 μM MG132 for 24 h. **p < 0.01 compared with the negative control. **h** The AGR2 promoter (0.5 or 1.4 kb) reporter constructs were transfected into A549 cells for 24 h, and then cells were treated with 5 μM MG132 for another 24 h. Luciferase activity was determined and was normalized to Renilla luciferase activity. ***p < 0.001 compared with the basic reporter

AGR2 protein is highly expressed in various human cancers including lung cancer [12], which is associated with poor patient survival [4] and also confirmed in our analysis (Fig. 1a). Consistent with the report [15], AGR2 was preferentially expressed in non-small cell lung cancer (NSCLC) H460 and A549 cells (Fig. 1b). When examining the changes of AGR2 in response to chemotherapeutic chemicals (data not shown), we found that proteasome inhibitor MG132 dose-dependently reduced AGR2 expression in A549 cells (Fig. 1c). It was noted that MG132 (~1 μM) effectively suppressed AGR2 expression, but had limited effect on cell proliferation and apoptosis (Fig. 1d, e), suggesting that the reduction of AGR2 might not be due to the MG132-mediated cell death. To validate the effect of MG132 on AGR2 expression that was dependent on proteasome inhibition, dynamic changes of p53 and p27, well-documented substrates of the proteasome, were examined in cells exposed to MG132. As shown in Fig. 1f, decreased AGR2 was observed at 0.5–2 h, and progressively declined up to 24 h during treatment. However, p53 and p27 were accumulated around 1 h, and maintained at high levels following prolonged treatments. Bortezomib, another proteasome inhibitor, exerted similar inhibitory effect on AGR2 expression (Fig. 1f). The results in Fig. 1g revealed that MG132 caused a remarkable decrease of the AGR2 mRNA. Promoter-based luciferase assays further confirmed that, compared with the control cells, MG132 dramatically suppressed the promoter activity of either 1.4 kb promoter or core promoter region in the AGR2 (Fig. 1h). These findings demonstrated that proteasome inhibition suppressed the expression of AGR2 both at mRNA and protein levels.

### Suppression of AGR2 by MG132 is independent of the induced ER stress and reactive oxygen species

**Fig. 2:**
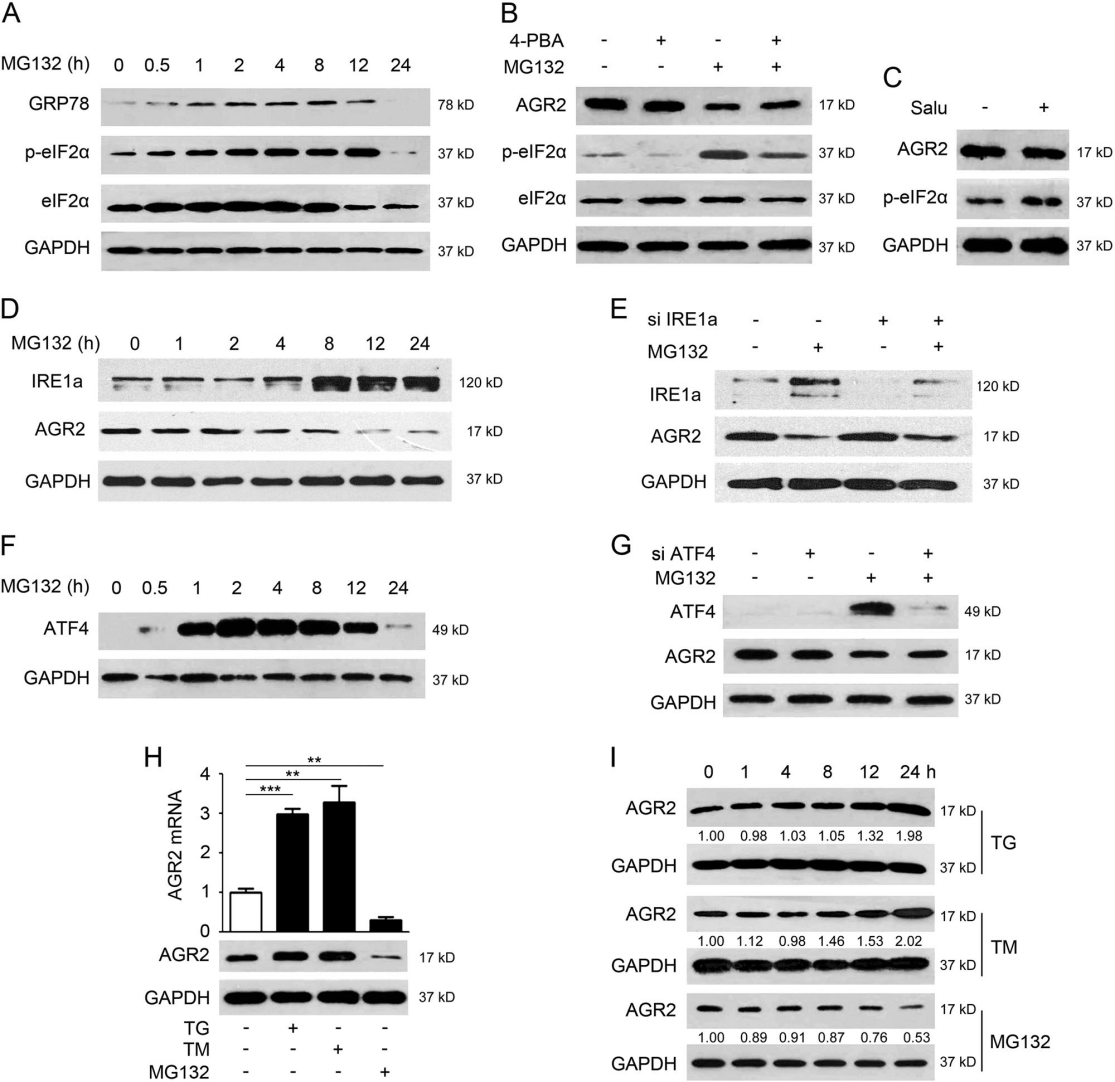
Suppression of AGR2 by MG132 is independent of the induced ER stress and ROS. **a** Analysis of the effect of MG132 on proteins associated with ER stress by western blot. **b** A549 cells were incubated with ER stress inhibitor 4-PBA (10 mM) for 2 h prior to MG132 (5 μM) treatment of 24 h. p-eIF2a and AGR2 expression were determined by western blot analysis. **c** The level of AGR2 was detected with 10 μM salubrinal treatment for 24 h. **d** The expression changes of IRE1a and AGR2 in MG132-treated A549 cells. **e** Knockdown of ATF4 by siRNA. **f** The expression change of ATF4 in 5 μM MG132-treated A549 cells was estimated by western blotting analysis. **g** Knockdown of ATF4 by siRNA. **h** QRT-PCR and western blotting analysis of mRNA and protein levels of AGR2 in TG (2 μM)/TM (2 mg/ml)/MG132 (5 μM)-treated A549 cells. GAPDH served as a loading control. **i** A549 cells were treated with ER stress inducer TG/TM/MG132 treatment at indicated time

ER stress response occurs when cells are challenged with accumulated proteins upon proteasome inhibition, we next checked if ER stress was responsible for the decrease of AGR2 upon MG132 treatment. As shown in Fig. 2a, the expression of glucose-regulated protein 78 (GRP78), a sensor of ER stress, increased markedly following a short exposure to MG132, and dropped down to the basal level after 12 h treatment. Similar to the change of GRP78, phosphor-eIF2α (inactive form) was evidenced at 0.5 h and gradually increased up to 12 h, whereas the total eIF2α almost remained unchanged during treatment. To test the importance of ER stress, changes in AGR2 were examined in the presence of 4-PBA that attenuates ER stress [16]. The results in Fig. 2b revealed that phosphor-eIF2α was decreased in response to 4-PBA, but AGR2 was not affected in cells exposed to 4-PBA alone or the combined treatments. Salubrinal, an eIF2α de-phosphorylation inhibitor, also had no effect on AGR2 abundance (Fig. 2c). As Higa et al. [13] report that AGR2 expression is up-regulated upon ER stress, which is controlled mainly by the ATF6a and IRE1a arms, changes of IRE1a were detected upon treatment. The results showed that MG132 increased IRE1a expression (same as PERK arm) at 8 h, at which decreased AGR2 was noted (Fig. 2d). However, knockdown of IRE1a did not resume AGR2 expression (Fig. 2e). We further assessed the contribution of ATF4, a critical transcription factor in regulation of ER stress-responsive gene expression. Induction of ATF4 was clearly observed upon MG132 treatment (Fig. 2f), but depletion of ATF4 did not affect AGR2 expression (Fig. 2g). In contrast to MG132, ER stress inducer thapsigargin (TG) and tunicamycin (TM) notably increased the AGR2 transcript and protein levels (Fig. 2h, i), implicating that the AGR2 acts as a chaperone in response to TG and TM, but not to MG132. qPCR analysis revealed that MG132 had limited effect on the expression of other members in PDI family (Sup.1A), supporting the specific response of AGR2 to MG132 treatment.

As AGR2 contains thioredoxin-like CXXS motifs with an active cysteine residue, we proposed that AGR2 expression might be responsive to reactive oxygen species (ROS) caused by MG132. However, blocking the ROS did not restore AGR2 expression as shown in Supplements (Sup. 1) Thus, MG132-triggered ER stress and ROS had limited effect on modulation of AGR2 expression.

### Repression of E2F1 by MG132 contributes to the inhibition of AGR2 transcription

**Fig. 3:**
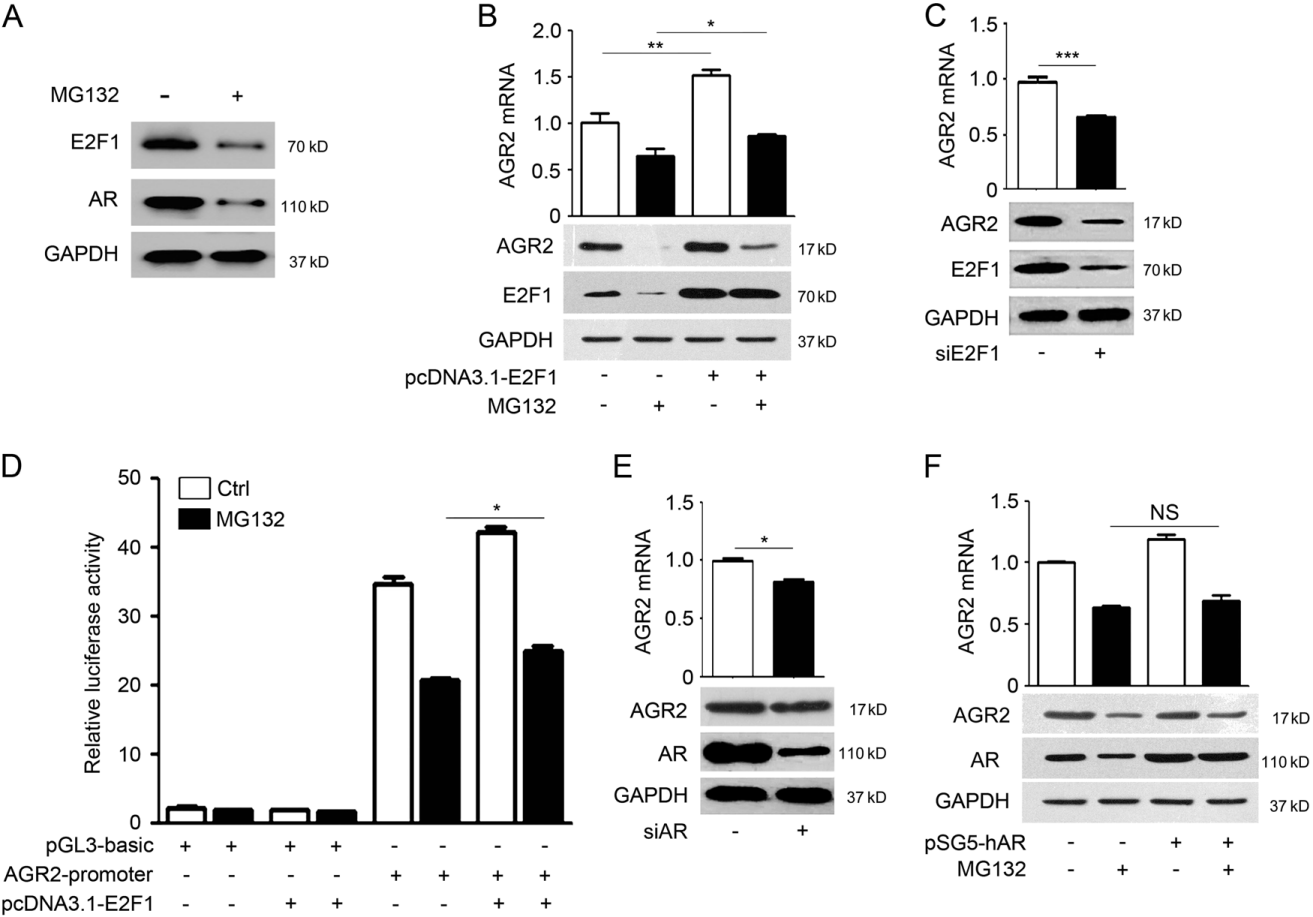
Repression of E2F1 by MG132 contributes to reduced AGR2 transcription. **a** The expression changes of E2F1 and AR in MG132-treated A549 cells were estimated by western blotting analysis. GAPDH served as a loading control. **b** A549 cells were transfected with E2F1 expression plasmid for 24 h prior to MG132 treatment. AGR2 mRNA and protein were investigated by QRT-PCR and western blotting analysis, *p < 0.05 and **p < 0.01 compared with the negative control. Data shown are the means ± SD, *n* = 3. **c** Impact of knockdown of E2F1 by siRNA on the AGR2 mRNA and protein. **d** The AGR2-promotor reporter constructs were co-transfected with E2F1 expression plasmid into A549 cells for 48 h prior to MG132 treatment. Luciferase activity was determined after MG132 treatment for 24 h and was normalized to Renilla luciferase activity. Data shown are the means ± SD, *n* = 3. **e** Knockdown of AR by siRNA in A549 cells. *p < 0.05. **f** The change of AGR2 mRNA and protein induced by overexpression of AR in MG132-treated A549 cell. Data shown are the means ± SD, *n* = 3

Hormones and several transcriptional factors, including androgen receptor (AR), FoxA1, and FoxA2, are implicated in positive regulation of AGR2 expression [12]. We found that treatment of cells with MG132 had no detectable effect on FoxA1 and FoxA2 expressions (data not shown), whereas the treatment significantly suppressed the AR and E2F1 protein (Fig. 3a), which coincide with reports that suggested a role of proteasome activity in regulating AR and E2F1 expression [17, 18]. Overexpression of E2F1 facilitated the AGR2 transcription, and partially restored AGR2 mRNA suppressed by MG132 (Fig. 3b), while downregulation of E2F1 significantly inhibited AGR2 expression (Fig. 3c). Luciferase activity analysis indicated that overexpression of E2F1 stimulated the AGR2 promoter reporter activity, and partially recovered AGR2 expression upon MG132 treatment (Fig. 3d). Regarding of the AR, downregulation of AR led to the reduced AGR2 (Fig. 3e), consistent with the previous report [19]. However, AR was unable to recover the AGR2 expression in the presence of MG132 (Fig. 3f), indicating that the impact of the AR on AGR2 expression was less significant compared to the E2F1. Therefore, reduction of E2F1 by MG132 played a role in suppression of the AGR2 transcription.

### MG132-stimulated autophagy is required for the degradation of AGR2

**Fig. 4:**
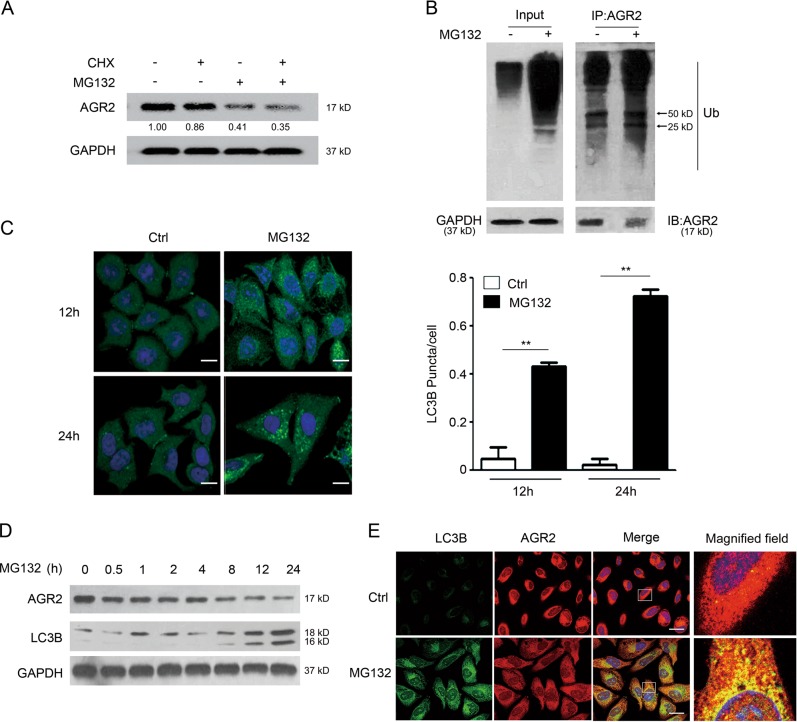
Activation of autophagy by MG132 is associated with AGR2 decline. **a** The expression change of AGR2 in A549 cells was estimated by western blotting analysis. A549 cells were incubated with CHX (2 μg/ml) for 2 h prior to MG132 treatment. GAPDH served as a loading control. **b** AGR2 ubiquitination was detected by immunoprecipitation with anti-AGR2 antibody and immunoblotting with anti-Ub antibody. Similar results were obtained with three independent experiments. **c** Immunofluorescence microscopy for analysis of punctate pattern of LC3 localization in A549 cells transiently transfected with the GFP-LC3-vector for 24 h and treated with MG132 for additional 12 or 24 h, and LC3 punctate (right panel) was calculated in A549 cells. **p < 0.01 compared with the control. Scale bar = 20 μm. **d** The levels of AGR2 and LC3B were measured by western blotting with MG132 treatment at indicated time. **e** Analysis of LC3B and AGR2 accumulation in A549 cells by fluorescent staining. Scale bar = 10 μm

To understand if the decreased AGR2 by MG132 is caused by protein stability, cycloheximide (CHX), a protein synthesis inhibitor, was included in the study below. The AGR2 abundance was reduced by CHX, and this decrease was further declined when MG132 was present (Fig. 4a). These results clearly displayed the involvement of AGR2 protein degradation following MG132 treatment.

Since proteasome inhibition results in accumulation of polyubiquitinated proteins, we performed immunoprecipitation assays to check if AGR2 is polyubiquitinated by MG132. As shown in Fig. 4b, an increase in the polyubiquitinated AGR2 was noticeably detected following MG132 treatment.

We next test if autophagy, which is activated and sequesters polyubiquitinated proteins for degradation when proteasome activity is suppressed [20–24], controls AGR2 expression. Exposure of cells to MG132 markedly caused accumulation of LC3B at 12 h and sustained up to 24 h as visualized by increased LC3B puncta in treated cells (Fig. 4c). The changes in the LC3B expression and the conversion of LC3B-I to lipidated LC3B-II further confirmed the autophagy activation by MG132 (Fig. 4d). Notably, the reduced AGR2 became more evident at the time points when the autophagic response was taking hold, suggesting that MG132-activated autophagy might be involved in the clearance of polyubiquitinated AGR2. Furthermore, co-staining by immunofluorescence with endogenous AGR2 and LC3 in A549 cells shown that MG132 caused the formation of large LC3-positive spots that were co-localized with AGR2 (Fig. 4e), supporting the observations that induction of autophagy by MG132 delivered AGR2 for degradation. Therefore, subsequent studies focused on the degradation of polyubiquitinated AGR2 protein by the autophagy–lysosome pathway.

**Fig. 5:**
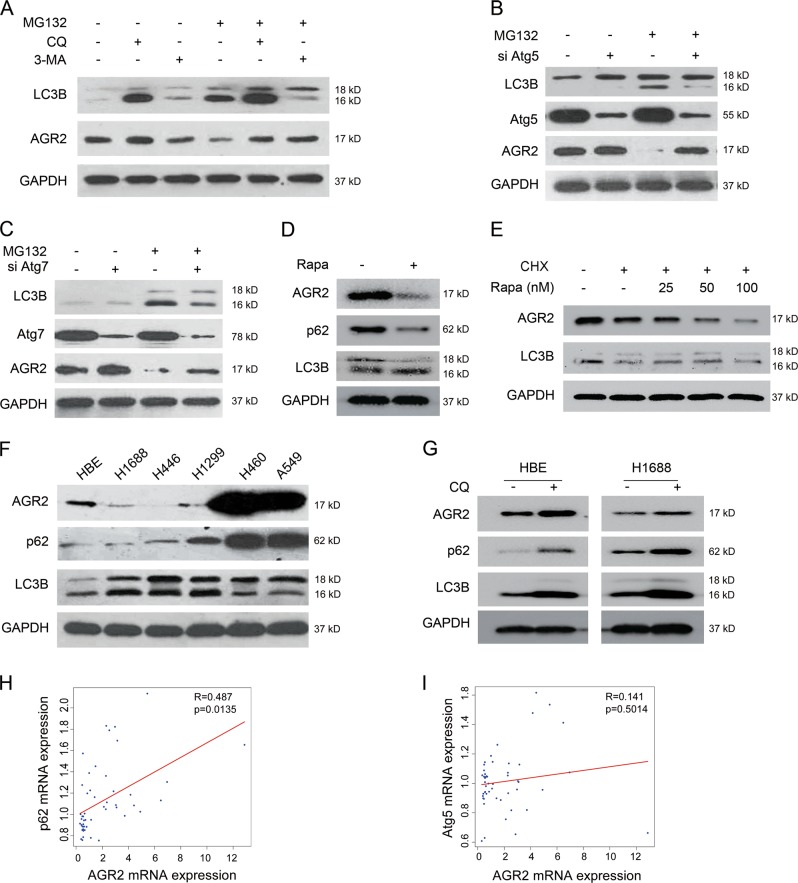
Autophagy stimulated by MG132 is required for the degradation of AGR2. **a** A549 cells were incubated with autophagy inhibitor 3-MA (5 mM) and CQ (10 μM) for 2 h prior to MG132 treatment. LC3B and AGR2 expression were determined by western blot analysis. **b**, **c** The expression changes of AGR2 in A549 cell, knockdown of **b** Atg5, and **c** Atg7 by siRNA for 48 h and then treated with MG132. **d** The level of AGR2 in Rapamycin (100 nM)-treated A549 cells. **e** A549 cells were treated with CHX and Rapamycin for 24 h. **f** The baseline expression of AGR2 and autophagy in normal bronchial epithelial cell line (HBE) and several lung cancer cell lines. GAPDH served as a loading control. **g** The expression changes of AGR2 with CQ treatment for 24 h. **h**, **i** The correlative analysis of AGR2 and p62/Atg5 in human lung cancer datasets (GSE27262) from GEO database was assessed by Pearson’s correlation test

Firstly, we co-treated cells with MG132 and 3-MA, an inhibitor of autophagic flux, to explore the role of autophagy in the clearance of AGR2. Inactivation of autophagy by 3-MA predominantly restored the AGR2 protein which was suppressed by MG132 (Fig. 5a). Similar changes of AGR2 were observed using chloroquine, another agent disrupting the autophagy maturation (Fig. 5a). Furthermore, we interrupted autophagy by genetic knockdown of Atg5 and Atg7, two crucial components involved in autophagy. As illustrated in Fig. 5b, c, depletion of Atg5 or Atg7 eliminated LC3B expression, and dramatically rescued MG132-mediated suppression of AGR2.

Reciprocally, activation of autophagy by rapamycin resulted in a decrease in AGR2 protein (Fig. 5d). Moreover, CHX caused the reduction in AGR2 was sharply declined in the presence of rapamycin (Fig. 5e), supporting the importance of autophagy in the clearance of AGR2. Driven by these observations, we next verified if there is a correlation between the AGR2 protein and autophagy in lung cancer cell lines. The results in Fig. 5f showed that AGR2 expression was significantly high in H460 and A549 cells, whose autophagy level, to some extent, was low as indicated by accumulation of p62 and reduction of LC3B-II/I. In contrast, the higher of LC3B-II/I was associated with the lower AGR2 in H1688, H446, and H1299 cells. Upon chloroquine treatment, the AGR2 was restored in HBE and H1688 cells (Fig. 5g).

The bioinformation analysis of AGR2 and p62 in public human lung cancer datasets from GEO database confirmed that there was a positive link between AGR2 and p62 (Fig. 5h), but AGR2 had no correlation with Atg5 (Fig. 5i). Thus, activation of autophagy by the proteasome inhibitor was responsible to the AGR2 protein degradation.

### Proteasome inhibition promotes ubiquitinated AGR2 autophagic degradation via cargo adaptor NBR1

**Fig. 6:**
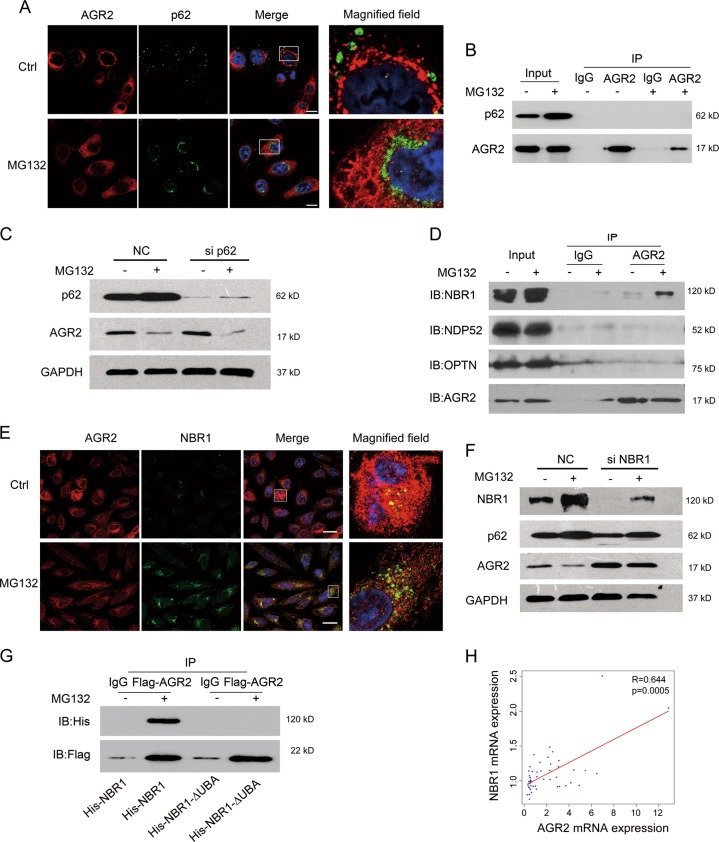
Proteasome inhibition promotes AGR2 autophagic degradation via cargo adaptor NBR1. **a** Immunofluorescence analysis of AGR2 and p62 localization treated by MG132 for 24 h in A549 cells. For confocal microscopy, AGR2 and p62 were immunostained, with nuclei stained with DAPI. Scale bar = 10 μm. **b** The interaction between AGR2 and p62 was evaluated in MG132-treated A549 cells by immunoprecipitation with anti-AGR2 antibody and immunoblotting (IB) with anti-p62 antibody. **c** Knockdown of p62 by siRNA, the change of AGR2 protein in MG132-treated A549 cells was analyzed by western blot. **d** The interaction between AGR2 and other autophagy receptors response to MG132 was evaluated by immunoprecipitation. **e** Immunofluorescence analysis of AGR2 and NBR1 localization. Scale bar = 10 μm. **f** Knockdown of NBR1 by siRNA, the change of AGR2 protein in MG132-treated A549 cells, was analyzed by western blot. **g** Flag-AGR2 and His-NBR1 or His-NBR1-ΔUBA (deletion of the ubiquitin-associated domain) were co-transfected into HEK293 cells, and the lysates from transfected cells were subjected to immunoprecipitation (IP) with anti-Flag antibody followed by immunoblotting (IB) analysis with anti-His antibody. **h** The Pearson’s correlation test was used to analyze the link between AGR2 and NBR1 from GEO database

We next determined whether p62, a cargo receptor for binding of ubiquitinated substrates [25, 26], was able to destine AGR2 for autophagic degradation. Immunofluorescence staining analysis indicated that AGR2 appeared to form aggresome-like dots in MG132-treated cells, while in control cells AGR2 was well distributed throughout the cytoplasm (Fig. 6a), consistent with the reports that polyubiquitinated proteins prone to form aggresomes triggered by prolonged proteasome inhibition [27, 28]. Surprisingly, p62 did not colocalize with AGR2 upon MG132 treatment (Fig. 6a). Co-immunoprecipitation revealed that p62 was unable to bind to AGR2 in MG132-treated cells (Fig. 6b). Furthermore, knockdown of p62 with siRNA did not alter AGR2 expression with or without MG132 (Fig. 6c), indicating that p62 may not contribute to the AGR2 degradation. We next determined the other autophagy receptor responsible for AGR2 degradation by co-immunoprecipitation assays [29, 30]. The results in Fig. 6d indicated that NBR1, but not NDP52 and OPTN, obviously bound to AGR2 in the presence of MG132. Co-staining by immunofluorescence with endogenous AGR2 and NBR1 confirmed that NBR1 overlaid with AGR2 in MG132-treated cells (Fig. 6e). Also, MG132-induced AGR2 degradation was markedly suppressed when NBR1 was downregulated by siRNA (Fig. 6f). As the ubiquitin-associated (UBA) domain of NBR1 binds both K48 and K63 linked polyubiquitin chains and regulates autophagic protein turnover [31], we found that full-length NBR1 was clearly present in immunocomplex precipitated by Flag-AGR2 upon MG132 treatment, whereas loss of UBA domain eliminated the interaction between NBR1 and AGR2 (Fig. 6g). Database analysis also presented a positive correlation between AGR2 and NBR1 (Fig. 6h), compared to that of p62 (Fig. 5h). Thus, these results demonstrated the involvement of NBR1 in AGR2 degradation by MG132.

### MG132 promotes K48-mediated AGR2 ubiquitylation via Lys89

**Fig. 7:**
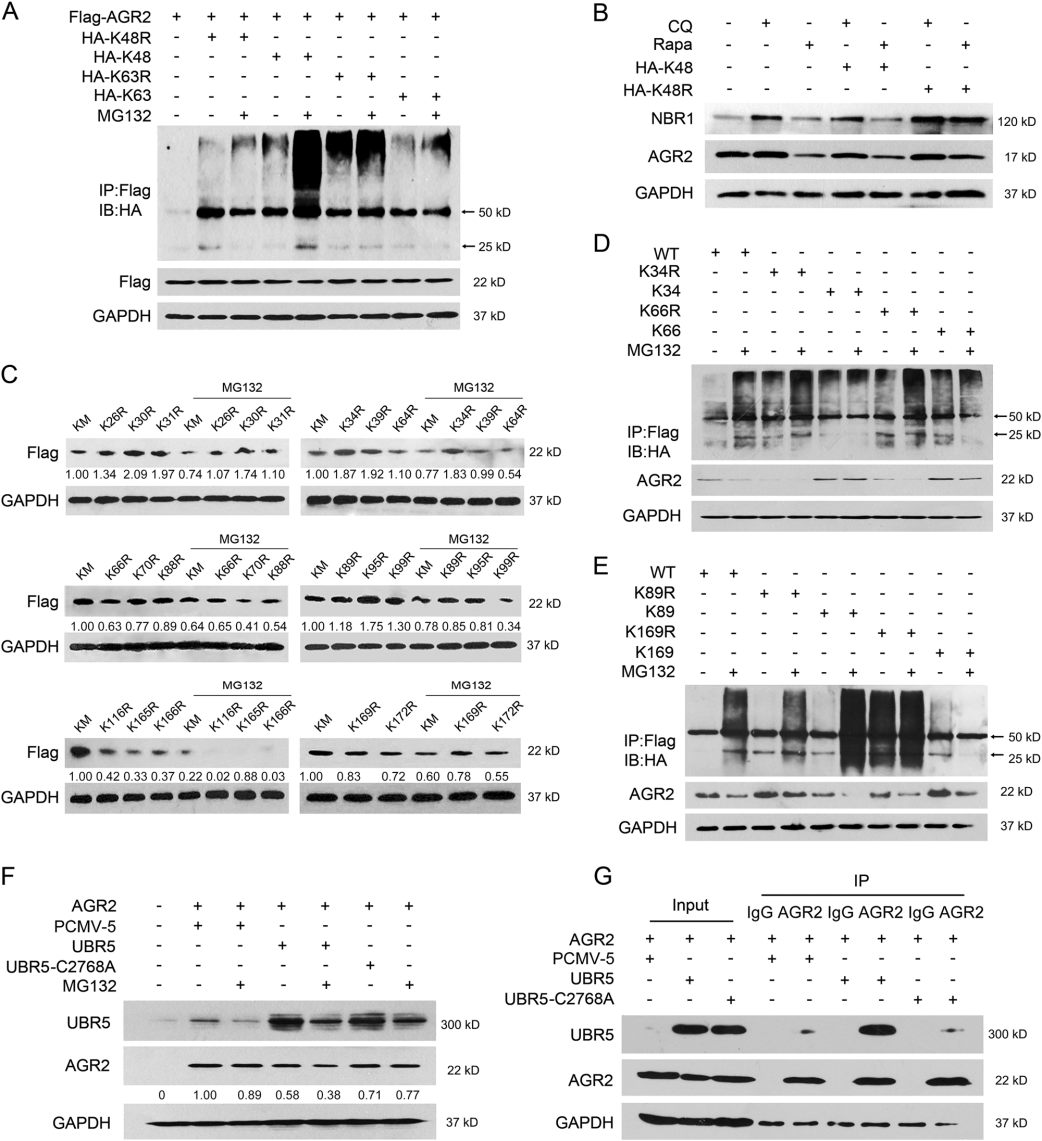
MG132 promotes K48-mediated AGR2 ubiquitylation via Lys89. **a** Plasmids encoding HA-K48 and K63 or mutants K48 (K48R) and K63 (K63R) were transfected into HEK293 cells together with Flag-AGR2. AGR2 ubiquitination in response to MG132 treatment for 8 h was detected by immunoprecipitation with anti-Flag antibody and immunoblotting with anti-HA antibody. **b** A549 cells transfected with HA-K48 or mutant K48 (K48R) expression plasmid were treated with autophagy inhibitor CQ (10 μM) or autophagy inducer Rapamycin (100 nM) for 12 h. The protein levels of AGR2 and NBR1 in the lysates were determined by immunoblotting. Similar results were obtained with three independent experiments. **c** HEK293 cells transfected with various Flag-AGR2 mutation plasmids (every lysine mutation plasmid and all lysine mutation plasmid Km were treated with MG132). The protein level of AGR2 was determined by immunoblotting. **d, e** Plasmids encoding Flag-AGR2 (WT) or mutants K34 (K34R)/K66 (K66R)/K89 (K89R)/K169 (K169R) were transfected into HEK293 cells together with HA-K48. AGR2 ubiquitination in response to MG132 treatment for 4 h was detected by immunoprecipitation with anti-Flag antibody and immunoblotting with anti-HA antibody. **f, g** HEK293 cells transfected with PCMV5-UBR5 or mutants (UBR5-C2768A) expression plasmid and Flag-AGR2 plasmid together were treated with MG132. **f** The protein level of AGR2 was determined by immunoblotting. **g** The interaction between AGR2 and UBR5 was evaluated by immunoprecipitation

As AGR2 was polyubiquitinated in response to MG132, ubiquitin mutant vectors containing K48, K63, K48R, and K63R (K-to-R substitution at position K48 or K63) were used to determine the form of ubiquitin chains linked to AGR2. The results showed that AGR2 was efficiently conjugated with ubiquitin containing K48 in the presence of MG132, with less affinity to K63 (Fig. 7a), indicating that MG132 induced K48-linked poly-ubiquitylation of AGR2. We transfected K48 or K48R in cells and examined the change of AGR2 in cells treated by rapamycin or chloroquine. As shown in Fig. 7b, rapamycin-activated autophagy facilitated AGR2 degradation in cells transfected with K48 mutant, but to less extent with K48R. In contrast, inactivation of autophagy by chloroquine attenuated K48-mediated AGR2 degradation (Fig. 7b). Therefore, MG132 led AGR2 to be polyubiquitinated preferentially via K48 ubiquitylation that was subsequently targeted for autophagic degradation.

There are seventeen lysine (K) residues in the AGR2 molecule (K26, K30, K31, K34, K39, K64, K66, K70, K88, K89, K95, K99, K116, K165, K166, K169, K172; Sup. 2), we then determined which Lys residue in AGR2 contributed to the ubiquitylation modified by MG132. As shown in Fig. 7c, transfection of a Km plasmid losing all Lys in the AGR2, to some extent, reversed the MG132-mediated reduction on AGR2. It appeared that there was no obvious reduction in AGR2 protein in cells transfected with K34R, K66R, K89R, or K169R mutant (single K-to-R substitution on K34, K66, K89, and K169 in AGR2) compared to other mutants in response to MG132 (Fig. 7c). Further validation demonstrated that expression of K34, K66, or K169 mutant (containing one Lys at the desired site) had limited regulatory effect on AGR2 ubiquitylation with or without MG132 (Fig. 7d, e). However, accumulation of polyubiquitinated AGR2 was clearly shown in cells transfected with AGR2 containing K89 in response to MG132 (Fig. 7e), indicating the K89 of AGR2 contributed to AGR2 ubiquitylation.

It has been reported that ubiquitin protein ligase E3 component-recognin 5 (UBR5) interacts with AGR2 [32]; we hypothesized that UBR5 may be the E3 ligase responsible for AGR2 ubiquitylation. As shown in Fig. 7f, increased expression of UBR5 significantly suppressed the AGR2 expression when exposed to MG132. In contrast, mutation of UBR5 at C2768A (a critical amino acid residue for ubiquitylation) markedly restored AGR2 upon proteasome inhibition (Fig. 7f). Immunoprecipitation assays confirmed that AGR2 noticeably bound to the UBR5, but had limited binding capability to mutant UBR5-C2768A (Fig. 7g). Therefore, involvement of the E3 ligase UBR5 was ascribed to K48-dependent AGR2 ubiquitylation.

### Suppression of AGR2 by proteasome inhibitor augments the antitumor efficiency of bevacizumab

**Fig. 8:**
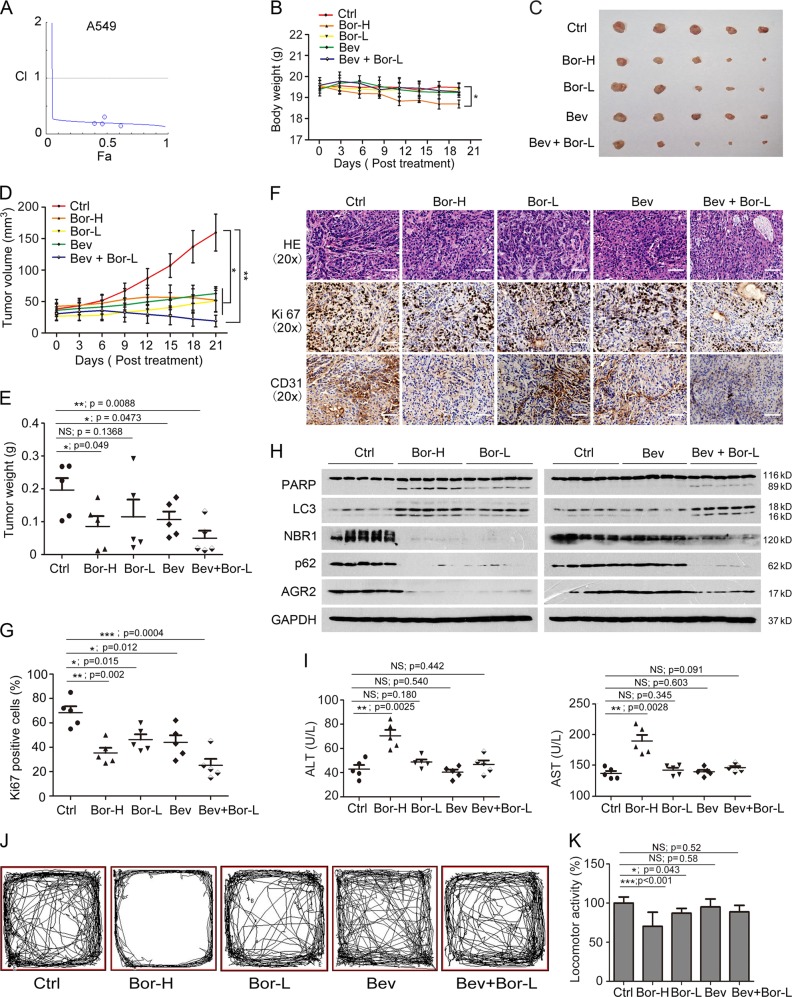
Proteasome inhibitor bortezomib augments the antitumor efficiency of bevacizumab in vitro and in vivo. **a** Combined effect of bortezomib and bevacizumab in A549 cells. After incubating cells with increasing concentrations of bortezomib and bevacizumab, cell viability was determined, and the combination index (CI) was calculated by CompuSyn software. **b** The mouse body weight of the five groups was measured every 3 days after the indicated treatment. **c** Representative tumors from the five groups are shown (*n* = 5). **d** Tumor volume from homografts in different treatment groups was recorded every 3 days. Data are represented as the mean ± S.E.M. (*n* = 5). **e** Tumor weight was detected at the time of sacrifice for different treated groups. **f** Representative images of H&E staining and immuno-histochemical staining of the different treated groups. Scale bar = 50 μm. **g** Ki67-positive rates in each group. *p < 0.05, **p < 0.01 and ***p < 0.001 compared with the negative control. Data are shown as the mean ± S.E.M. **h** Western blot analysis of AGR2, PARP, LC3 expression in differentially treated groups. **i** As liver function tests, ALT and AST were examined at the time of sacrifice for different treated groups. Data are shown as the mean ± S.E.M (*n* = 5). **j**, **k** Locomotor activity of different treated groups was detected at 24 h after each injection. Data are shown as the mean ± S.E.M (*n* = 15). **p < 0.01 compared with the negative control

Clinical trials of bortezomib in combination with bevacizumab (Bev), our previous study has revealed that secreted AGR2 directly binds to VEGF and enhances VEGFR2 signaling, leading to the interruption of antitumor activity of bevacizumab, a humanized monoclonal antibody that inhibits VEGF activity and has been approved for patients with cancers [10]. We reasoned that downregulation of AGR2 by bortezomib could be a mechanism conferring the enhanced antitumor efficiency of bevacizumab. The results in Fig. 8a and Sup. 3 showed that bortezomib synergistically increased the inhibitory activity of bevacizumab on A549 cell proliferation, but not H1299. Based on the high toxicity of bortezomib, we designed an animal study to test the possibility that low dose of bortezomib (Bor-L) enhanced the antitumor efficiency of bevacizumab, associated with the suppression of AGR2 and low toxicity in mice developed from A549 cells in which AGR2 is overexpressed. The results in Fig. 8b revealed that, compared to the placebo group, body weight of mice treated with high dose of bortezomib (Bor-H) significantly decreased, whereas remained almost unchanged in other groups.

After treatments, bortezomib^high^ exerted better activity than that of bortezomib^low^, as evidenced by the changes in tumor size and weight (Fig. 8c–e). Of note, combined treatments with bortezomib^low^ and bevacizumab displayed the potential antitumor efficiency on mice, which was comparable, or better to the group treated with bortezomib^high^ or bevacizumab alone (Fig. 8c–e). Also, the combined treatment predominantly eliminated the positive Ki67 staining cells compared to bevacizumab treatment alone (Fig. 8f, g). Interestingly, interruption of angiogenesis by bevacizumab was also enhanced in the presence of bortezomib^low^ as evidenced by decreased CD31 positive staining (Fig. 8f). Molecular marker analyses demonstrated that both high and low dose of bortezomib noticeably suppressed AGR2 abundance (Fig. 8h). The change pattern of autophagy markers, including LC3B-II/LC3B-I, p62, and NBR1, was consistent with the observations in cultured cells (Fig. 8h). In addition, upon bevacizumab treatment, no significant changes were observed in PARP cleavage, activation of autophagy, and AGR2 abundance (Fig. 8h) However, combination of bevacizumab with bortezomib^low^ resulted in an increased PARP cleavage and a reduced AGR2, which was associated with activation of autophagy (Fig. 8h). The combined treatment caused limited toxicity in mice, as ALT and AST levels remained unchangeable compared to the group treated with bevacizumab or bortezomib^high^ alone (Fig. 8i). Moving distance of mice decreased in each group after treatments, particularly in the bortezomib^high^ group (Fig. 8j, k). Also, combined treatment did not affect the intake of food and water in mice compared to other treated groups (Sup. 3). Therefore, AGR2 may be a predictive marker for selection of patient group that is suitable for combination therapy with bevacizumab and bortezomib with high efficiency and low toxicity.

## Discussion

In the present study, we provided a novel explanation that proteasome inhibition resulted in a significant decrease of AGR2 in cancer cells. The reduced AGR2 was partially a consequence of the decreased mRNA level mediated by downregulation of E2F1. Activation of autophagy, but not the ER stress activation or ROS generation, further facilitated the clearance of polyubiquitinated AGR2 protein due to proteasome inhibition. We also demonstrated that proteasome inhibition-mediated poly-ubiquitylation of the AGR2 was mainly dependent on the formation of K48-linked polyubiquitin chains on K89 of AGR2. E3 ligase UBR5 contributed to the polyubiquitination of AGR2, and autophagy receptor NBR1 was required for recruiting polyubiquitinated AGR2 for autophagic clearance. The effect of proteasome inhibitor on abolishment of AGR2 enhanced the antitumor activity of bevacizumab.

Besides its proteolytic activities, proteasome also provides non-proteolytic functions involved in multiple aspects of transcription- and translation-related processes [17].

Our study showed that proteasome inhibition is able to suppress AGR2 transcription by downregulation of E2F1. It cannot rule out a possibility that other transcription factor(s) including FOXA1/2 [12], FOXP1/4 [33], SOX10 [34], and ZNF217 [35] is involved in transcriptional regulation of AGR2 expression in response to MG132. Further investigation is required to elucidate the mechanisms that proteasome inhibition transcriptionally regulates AGR2 expression. Suppression of the AGR2 by proteasome inhibition has been attributed to the induction of autophagy, instead of triggering ER stress and global translation repression. There was a strong correlation between AGR2 and p62 expression levels among human lung cell lines and clinical sample data presented in GEO database. This notion may provide an additional explanation why AGR2 expression is relative high in human lung cancers where impaired autophagy was observed [3, 36]. Additionally, our data revealed that the ubiquitylation of AGR2 was K48 ubiquitin chain linkages. It has been proposed that K48-linked ubiquitin chains may preferentially allow proteins to undergo proteasome degradation [37]. Some reports provide evidence that K48-linked ubiquitin chains also can be recognized by cargo receptors, for example, p62 and NBR1. We found that the C-terminal UBA domain of NBR1 was crucial for binding to the ubiquitinated AGR2 for autophagic degradation, consistent with the reports that NBR1 protein binds ubiquitin via the UBA domain for selective autophagic clearance of ubiquitinated substrates [38].

We recently explored a novel mechanism by which extracellular AGR2 bound to VEGFA, leading to enhancement of VEGFR signaling. The pro-angiogenic and pro-metastatic activity of AGR2 caused less response of tumor to bevacizumab treatment [10]. The unique regulatory effect of proteasome inhibitors on AGR2 encouraged us to test a possibility that bortezomib enhances antitumor activity of bevacizumab. As expected, bortezomib at a very low concentration significantly augmented efficiency of bevacizumab, and lowered cytotoxicity in mice. We noted that the combination of bevacizumab and bortezomib did not result in significant improvements in efficacy outcomes in unselected patients with pretreated multiple myeloma [39], but was well tolerated with interesting clinical activity in treatment of NSCLC [40]. Our finding indicated that AGR2 might present a mechanism to affect outcomes of combination bevacizumab and bortezomib in patients. Further clinical study is required to define the predictive potential of AGR2 in cancer therapy.

In conclusion, the novel finding of this study is to explore the regulatory mechanism by proteasome inhibitors on AGR2 expression, particularly to explain the AGR2 ubiquitylation and autophagic degradation in cancer cells. Importantly, we provided evidence that bortezomib augments efficiency of bevacizumab by downregulation of AGR2, which may be a useful predictive marker for selection of subgroup patients in chemotherapy.

## Materials and methods

### Survival and Pearson correlation analysis

A newly developed interactive web server called GEPIA, which contains RNA sequencing expression data of 9736 tumors and 8587 normal samples from the TCGA and the GTEx projects [41], was used for analysis of AGR2 mRNA expression in lung adenocarcinoma. After calculation of median expression levels of AGR2 in all lung adenocarcinoma samples and paired normal tissues, the difference in overall survival between patience with lung adenocarcinoma with higher and lower than median AGR2 expression was subsequently calculated. Gene expression data (GSE27262, 25 normal samples and 25 tumors) retrieved from Gene Expression Omnibus (http://www.ncbi.nlm.nih.gov/geo), after data interpretation and normalization, Pearson correlation coefficient (*R*) between AGR2 and autophagy-related genes was analyzed using cor function in R programming language, *p* value is calculated using Pearson Calculator. *p* < 0.05 is considered significantly.

### Cells and chemicals

Human A549, H1299, H1688, H446 cells, and a human normal bronchial epithelial cell line (HBE) were purchased from Shanghai Cell Library of Chinese Academy of Science. MG132, Chloroquine (CQ), 3-[4,5-dimethylthiazol-2-yl]-2,5-diphenyltetrazoliumbromide (MTT), Thapsigargin (TG), CHX, and 3-methyladenine (3-MA) were purchased from Sigma-Aldrich. Tunicamycin (TM) and actinomycin D were obtained from Solarbio. Sodium 4-phenylbutyrate (4-PBA) was from Abcam. *N*-acetyl-l-cysteine (NAC) was from Beyotime. Rapamycin (Rapa) and salubrinal were purchased from Calbiochem and Santa Cruz, respectively.

### Quantitative PCR

Total RNA was extracted from cultured cells using an RNAiso plus kit (TaKaRa) according to the manufacturer’s instructions. The cDNA was synthesized from RNA using Prime Script™ RT reagent Kit (TaKaRa). Quantitative PCR (qPCR) analysis was performed on a real-time PCR system (Eppendorf International) using SYBR Green reaction master mix (TaKaRa). The desired gene expressions were normalized to the level of glyceraldehyde-3-phosphate dehydrogenase (GAPDH) and changes were calculated by the ΔΔCt method. Primer sequences are available in Supplementary Table 1.

### Plasmids

The human gene AGR2 was cloned into the pmCherry-C1 expression vector (Clontech). The AGR2 promoter region −1267 to +126 (~1.4 kb), or −372 to +126 (~0.5 kb) was amplified from human genomic DNA by PCR. The report constructs containing 0.5 kb (pGL3-AGR2–0.5 kb) and 1.4 kb (pGL3-AGR2–1.4 kb) sequence were obtained and verified after insertion of the fragments into pGL3-basic reporter vector (Promega). The E2F1 and GFP-LC3B expression plasmids were from Srikumar P. Chellappan (Lee Moffitt Cancer Center and Research Institute, Tampa, USA) [42]; His-NBR1 or His-NBR1-ΔUBA expression plasmids were purchased from Addgene and Xuejun Jiang (Memorial Sloan-Kettering Cancer Center, New York, NY, USA) [43], respectively. Expression vectors for HA-K48, HA-K48R, HA-63, and HA-K63R were from Chengjiang Gao (Department of Immunology, School of Medicine of Shandong University, China) [44]. AGR2 and its site-directed mutations expression plasmids were generated by means of two steps PCR and subcloned into pCDNA3.1-3xflag expression vector and listed in Supplementary Table 2 in detail. All constructs were confirmed by DNA sequencing.

### Transient transfection

Cells were transfected with plasmids or siRNA oligonucleotides using Lipofectamine2000 (Invitrogen). For Luciferase reporter assays, the pRL-TK renilla luciferase was served as a normalizing control. One day after transfection, cells were treated with MG132 for an additional 2 h, and cell lysates were subjected to dual luciferase assays (Promega). For RNA interference, siRNA duplex oligonucleotides or scramble oligonucleotides were synthesized (Invitrogen). In total, 100 pmol siRNA was added in one well from a six-wells plate. The siRNA sequences are provided in Supplementary Table 3.

### Western blotting and immunoprecipitation

Cells were lysed in RIPA buffer supplemented with protease inhibitors and phosphatase inhibitors (Invitrogen). Protein concentrations were quantified by BCA assay (Beyotime). Samples containing equal amounts of protein were electrophoresed as previously reported [2]. The membranes were probed with following antibodies: LC3B (Novus, NB600–1384), AGR2 (Abcam, ab209224), (GeneTex, gt5812), Ub (Abcam, ab19247), Flag (Sigma, F1804), HA (Sigma, H9658), p62 (Santa, sc-28359), NBR1 (Santa, sc-130380), IRE1a (Santa, sc-390960), glucose-regulated protein 78 (GRP78)(Santa, sc-376768), ATF4 (Santa, sc-200), p27 (Santa, sc-56338), p53 (Santa, sc-71820), AR (Santa, sc-7305), E2F1 (sc-251), GAPDH (Santa, sc-47724), NDP52 (Proteintech, 12229-1-AP), OPTN (Proteintech, 10837-1-AP), Atg5 (CST, 9980), Atg7 (CST, 2631), p-P65 (CST, 3033p), phospho-eIF2α (Ser51) (CST, 9721), and eIF2α (CST, 5324). For immunoprecipitation, cell lysates were pre-cleared with protein G plus-Agarose (Santa Cruz), and then incubated with 1 μg anti-AGR2, control IgG (Santa Cruz), or anti-Flag on a rotary mixer at 4 °C overnight. The immunocomplexes were captured by addition of 20 μl protein G plus-agarose beads, and washed three times with RIPA buffer. After recovery by heating at 95 °C for 5 min in 20 μl eluent with 0.1 M glycine (pH 3.0) twice, samples were neutralized by 1 M Tris-HCl of pH 8.5. After mixed with 4× SDS loading buffer, the immunocomplexes were analyzed by western blotting.

### Immunofluorescence assay

Cells grown on coverslips were washed twice with phosphate-buffered saline (PBS), then fixed with ice-cold methanol/acetone (1:1) for 5 min, and incubated with 3% bovine serum albumin (BSA) for 20 min. Following rinsed with PBS, coverslips were treated with the primary antibodies at 4 °C overnight. Images were acquired by a confocal microscopy (Carl Zeiss) after incubation with secondary antibodies and DAPI.

### ROS detection and cell viability assay

Cellular ROS concentrations were measured with a flow cytometer (BD Biosciences) after incubating cells with a fluorescence probe 2,7-dichlorofluorescein-diacetate (DCFH-DA; Sigma-Aldrich). Cell viability was determined via a 3-(4,5-dimethylthiazol-2-yl)-2,5-diphenyl-2H-tetrazoliumbromide (MTT, Sigma) assays on a plate reader (Bio-Rad, Hercules, CA, USA).

### Drug combination analysis

CalcuSyn (Biosoft, Ferguson, MO) software was used for evaluation of interaction between drugs [45]. This program uses the median effect analysis algorithm, which produces the combination index (CI) value as a quantitative indicator of the degree of synergy or antagonism. CI < 1, CI = 1, and CI > 1 indicate synergistic effect, additive effect, and antagonistic effect, respectively.

### Effect of proteasome inhibitor on antitumor efficiency of bevacizumab in vivo

Lung cancer A549 cells were inoculated subcutaneously into the right flank region of male athymic (BALB/c-nu) mice (6–8 weeks old). When tumor size reached approximately 100 mm^3^, 25 mice were randomized block design by tumor size and divided into five groups that treated by intraperitoneal injections of vehicle saline containing 1% DMSO (Ctrl group), 0.8 mg/kg bortezomib (Bor-H group), 0.4 mg/kg bortezomib (Bor-L group), 10 mg/kg bevacizumab (Bev group), or 0.4 mg/kg bortezomib and 10 mg/kg bevacizumab (Bev + Bor-L group). The injection was performed every weekly for three consecutive weeks. The animal weight, tumor volume, and feed intake were recorded. The locomotor activity test was performed at 24 h after each injection to evaluate the drug for safety, as previously reported [46]. The tumor volume (in mm^3^) was calculated from the formula 0.5 × *L* × *W*^2^ (*L* = length, *W* = width). At the end of the experiment, the whole blood was collected from mice orbit for liver function tests, and all tumors were resected for western blotting and immunohistochemistry. All animal experiments were approved by the Ethics Committee of Shandong University School of Medicine (Permit Number: LL-201602042) and conducted accordingly.

### Statistical analysis

Kaplan–Meier and log-rank test were used for survival analysis. The data are presented as the mean ± SD of at least three independent experiments. One-way ANOVA with Bonferroni’s post-test was used to compare multiple group comparisons. The difference between the control and treated groups was determined with Student’s *t*-tests.

## Supplementary information

Supplementary data
