# Supplementary material for: Proteasome inhibition boosts autophagic degradation of ubiquitinated-AGR2 and enhances the antitumor efficiency of bevacizumab
Source: Oncogene. 2019 Jan 15;38(18):3458–74. doi: 10.1038/s41388-019-0675-z (PMC6756021; doi:10.1038/s41388-019-0675-z)
Supplement: Supplementary file 1 — Supplementary data [file 41388_2019_675_MOESM1_ESM.pdf]

Proteasome inhibition boosts autophagic degradation of ubiquitinated-AGR2 and enhances the antitumor efficiency of bevacizumab

Dawei Wang[1#] (master), Qingqing Xu[1#] (doctor), Quan Yuan[1#] (master), Mengqi Jia[1] (doctor), Huanmin Niu[1] (doctor), Xiaofei Liu[1] (master), Jinsan Zhang[2] (doctor), Charles YF Young[2] (doctor), Huiqing Yuan[1*] (doctor)

[1] Key Laboratory of Experimental Teratology of the Ministry of Education, Institute of medical sciences, the Second Hospital of Shandong University, Jinan, China

[2]Department of Urology, Mayo Clinic College of Medicine, Mayo Clinic, Rochester, MN, USA

Running title: proteasome inhibition reduces AGR2 expression

Conflict of interest

The authors declare no conflict of interest

[#]These authors contributed equally: Dawei Wang, Qingqing Xu, Quan Yuan

*Correspondence: Institute of medical sciences, the Second Hospital of Shandong University, 247 Beiyuan Dajie Street, Jinan 250033, China. Phone: 86-531-85875027. E-mail: lyuanhq@sdu.edu.cn (H.Yuan).

Supplementary Information

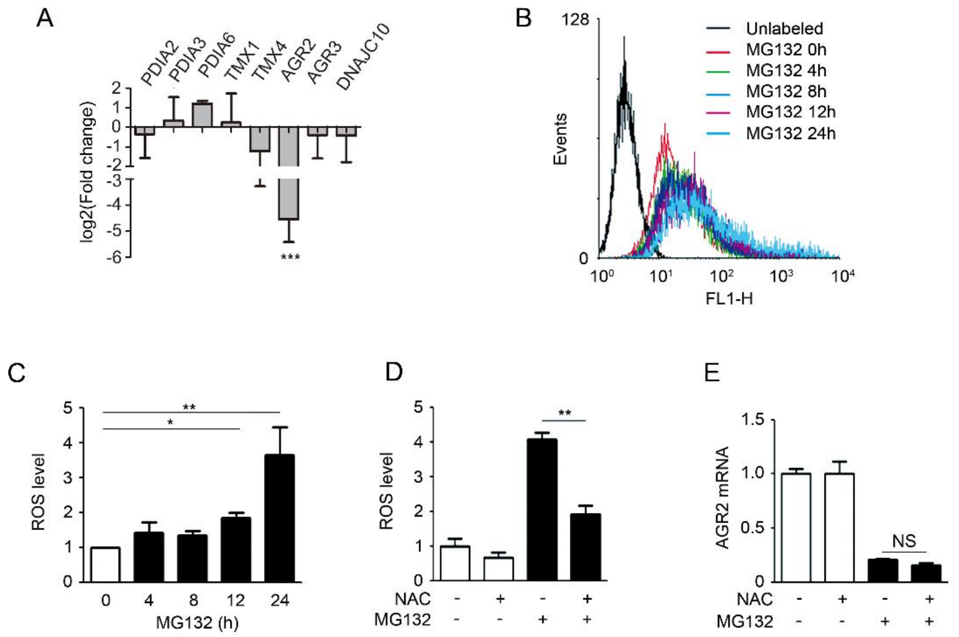

Sup.1 Suppression of AGR2 by MG132 is independent of the induced ER stress and ROS. (A) The gene expression change of protein disulfide isomerase family induced by MG132. (B) Effect of 5μM MG132 on intracellular ROS formation in A549 cells assayed by flow cytometry stained with H(2)DCFDA at different time points and (C) the means of ROS level was calculated. (D) The levels of ROS and (E) the AGR2 mRNA were detected in A549 cells incubated with NAC (5mM) for 2h prior to MG132 treatment.

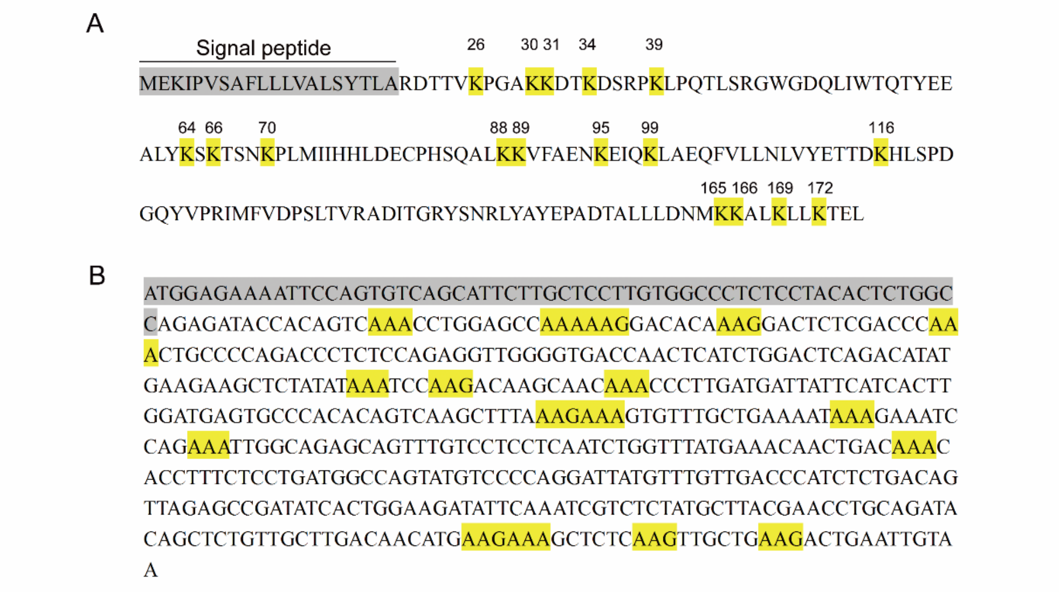

Sup.2 (A)The protein sequence and (B) the cDNA sequence of AGR2

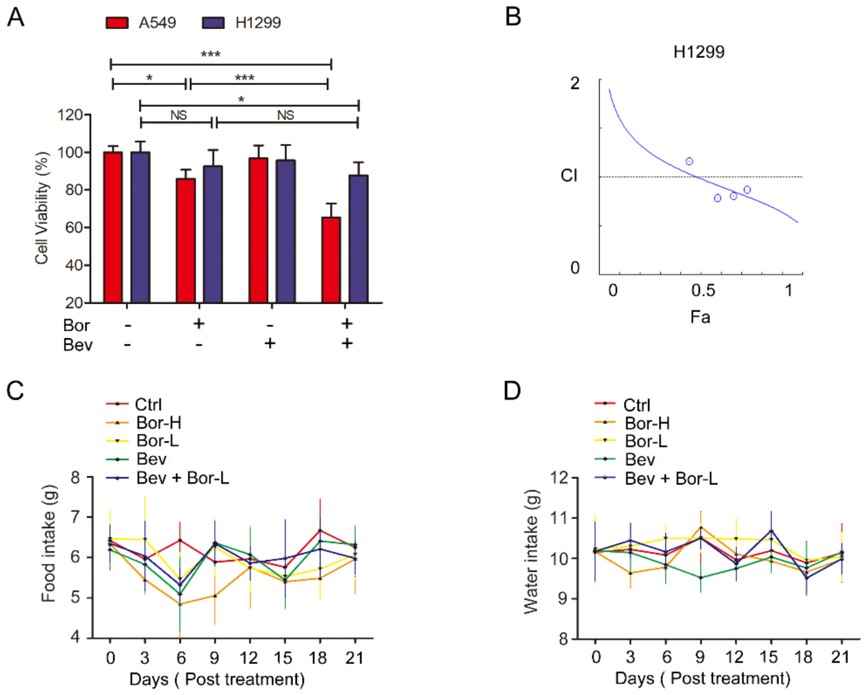

Sup.3 (A) Combined used of bortezomib and bevacizumab in A549 and H1299 cells. (B) After incubating cells with increasing concentrations of bortezomib and bevacizumab, cell viability was determined, and the combination index (CI) of H1299 cells was calculated by CompuSyn software. (C) (D)The intake of food and water were recorded every 3 days after the indicated treatment.

Supplementary table 1. RT-PCR Primers

Gene	Forward	Reverse
PDIA2	GCGTCCATCACTGCTTTCTG	TTGCCCACGAGGGTCTTAAC
PDIA3	TGCCCTCACATGACAGAAG	TTGCCACCATCATTACCCTG
PDIA6	CAATGCACGCAAGATGAAA	CAACGATGGTAGGGAAAGC
TMX1	GCAGATTGCCTTTGTCCTTC	TCTTCATCCGCCTCTTGTTC
TMX4	TGGACGCTGGTGATGGAG	CTCAAACCTGGTTCTTGAATG
AGR2	GCATTCTTGCTCCTTGTGG	AGGGTTTGTTGCTTGTCTTG
AGR3	GAGATGACATCACTTGGGTACA	GCATGATGAACTTATTCTGAGCC
DNAJC10	AGCTATCCCAGCCTCTTCAT	CAGCCAATACCAGCAGCAA
GAPDH	GCCTCAAGATCATCAGCAATG	GTCAAAGGTGGAGGAGTGG

Supplementary table 2. Primers for HA-AGR2 mutations

Plasmid	Forward	Reverse
K26R	GAGATACCACAGTCAGACCTGGAGCCAAAAAG	CTTTTTGGCTCCAGGTCTGACTGTGGTATCTC
K30R	CAAACCTGGAGCCAGAAAGGACACAAAG	CTTTGTGTCCTTTCTGGCTCCAGGTTTG
K31R	CTGGAGCCAAAAGGGACACAAAGGAC	GTCCTTTGTGTCCCTTTTGGCTCCAG
K34R	CCAAAAAGGACACAAGAGACTCTCGACCCAAAC	GTTTGGGTCGAGAGTCTCTTGTGTCCTTTTTGG

K39R	AAAGGACTCTCGACCCAGACTGCCCCAGA	TCTGGGGCAGTCTGGGTCGAGAGTCCTTT
K64R	GAAGAAGCTCTATATAGGTCCAAGACAAGCAAC	GTTGCTTGTCTTGGACCTATATAGAGCTTCTTC
K66R	CTCTATATAAATCCAGGACAAGCAACAAACCC	GGGTTTGTTGCTTGTCCTGGATTTATATAGAG
K70R	CCAAGACAAGCAACAGGCCCTTGATGATTATTC	GAATAATCATCAAGGGCCTGTTGCTTGTCTTGG
K88R	CAGTCAAGCTTTAAGGAAAGTGTTTGCTG	CAGCAAACACTTTCCTTAAAGCTTGACTG
K89R	GTCAAGCTTTAAAGCGAGTGTTTGCTGAAA	TTTCAGCAAACACTCGCTTTAAAGCTTGAC
K95R	GTGTTTGCTGAAAATCGCGAAATCCAGAAATTGG	CCAATTTCTGGATTTCGCGATTTTCAGCAAACAC
K99R	GAAAATAAAGAAATCCAGCGCTTGGCAGAGCAGTTTGTC	GACAAACTGCTCTGCCAAGCGCTGGATTTCTTTATTTTC
K116R	GAAACAACTGACAGACACCTTTCTCCTG	CAGGAGAAAGGTGTCTGTCAGTTGTTTC
K165R	GAAGAAAGCTCTCAGGTTGCTGAAGACTG	CTTGAGAGCTTTCCTCATGTTGTCCTTG
K166R	GACAACATGAAGAGAGCTCTCAAGTTGC	GCAACTTGAGAGCTCTCTTCATGTTGTC
K169R	GAAGAAAGCTCTCAGGTTGCTGAAGACTG	CAGTCTTCAGCAACCTGAGAGCTTTCTTC
K172R	CTCAAGTTGCTGAGGACTGAATTGTAA	TTACAATTCAGTCCTCAGCAACTTGAG
K26	GATACCACAGTCAAACCTGGAGCCAG	CTGGCTCCAGGTTTGACTGTGGTATC
K30	GACCTGGAGCCAAGAGGGACACAAG	CTTGTGTCCCTCTTGGCTCCAGGTC
K31	CTGGAGCCAGAAAAGACACAAGGGAC	GTCCCTTGTGTCTTTTCTGGCTCCAG
K34	GAAGGGACACAAAAGACTCTCGACC	GGTCGAGAGTCTTTTGTGTCCCTTC
K39	ACTCTCGACCCAAACTGCCCCAGAC	GTCTGGGGCAGTTTGGGTCGAGAGT
K64	GAAGAAGCTCTATATAAGTCCAGGACAAGCAAC	GTTGCTTGTCCTGGACTTATATAGAGCTTCTTC
K66	CTCTATATAGATCCAAGACAAGCAACAGACC	GGTCTGTTGCTTGTCTTGGATCTATATAGAG
K70	GATCCAGGACAAGCAACAAACCCTTGATGATTATTC	GAATAATCATCAAGGGTTTGTTGCTTGTCCTGGATC
K88	CAGTCAAGCTTTAAAGAGAGTGTTTGCTG	CAGCAAACACTCTCTTTAAAGCTTGACTG
K89	CACAGTCAAGCTTTAAGGAAGGTGTTTGCTGAAAATAGAG	CTCTATTTTCAGCAAACACCTTCCTTAAAGCTTGACTGTG
K95	GTGTTTGCTGAAAATAAGGAAATCCAGAGATTGGC	GCCAATCTCTGGATTTCCTTATTTTCAGCAAACAC
K99	GAGAAATCCAGAAATTGGCAGAGCAG	CTGCTCTGCCAATTTCTGGATTTCTC
K116	GAAACAACTGACAAACACCTTTCTCCTG	CAGGAGAAAGGTGTTTGTCAGTTGTTTC
K165	CTTGACAACATGAAGAGAGCTCTCAGG	CCTGAGAGCTCTCTTCATGTTGTCAAG
K166	GACAACATGAGGAAGGCTCTCAGGTTG	CAACCTGAGAGCCTTCCTCATGTTGTC
K169	GGAGAGCTCTCAAATTGCTGAGGACTG	CAGTCCTCAGCAATTTGAGAGCTCTCC
K172	CTCAGGTTGCTGAAGACTGAATTGGAC	GTCCAATTCAGTCTTCAGCAACCTGAG

Supplementary table 3. The sequences of siRNA

Name	Sense	Antisense
E2F1 siRNA	AUGCUACGAAGGUCCUGACACGUCA	UGACGUGUCAGGACCUUCGUAGCAU
ATF4 siRNA	UCAUCUAAGAGACCUAGGCTT	GCCUAGGUCUCUUAGAUGATT
Atg5 siRNA	CCAUCAAUCGGAAACUCAUTT	AUGAGUUUCCGAUU GAUGGTT
Atg7 siRNA	GGUCAAAGGACGAAGAUAATT	UUAUCUUCGUCCUUUGACCTT
P62 siRNA	GGAACAGAUGGAGUCGGAUTT	AUCCGACUCCAUCUGUUCCTT
NBR1 siRNA	GCGCUUAAGAUGGCAGUUATT	UAACUGCCAUCUUAAGCGCTT
IRE1a siRNA	UUACUGGCUUCUGAUAGGATT	UCCUAUCAGAAGCCAGUAATT

Scramble siRNA	UUCUCCGAACGUGUCACGUTT	ACGUGACACGUUCGGAGAATT
